# The relationship between advanced glycation end products, metabolic metrics, HbA_1c,_ and diabetic nephropathy

**DOI:** 10.3389/fendo.2025.1468737

**Published:** 2025-03-07

**Authors:** Liping Xue, Yi Zhang, Qiu Zhang

**Affiliations:** Department of Endocrinology, First Affiliated Hospital of Anhui Medical University, Hefei, China

**Keywords:** advanced glycation end products, BMI, diabetes, UACR, obesity, TyG-BMI

## Abstract

**Background:**

In this cross-sectional study, we aim to investigate the value of non-invasive advanced glycation end products (AGEs) detection in the early screening of diabetic nephropathy(DN) among individuals with type 2 diabetes mellitus and assess whether metabolic parameters and glycated hemoglobin A_1c_ (HbA_1c_) can moderate this relationship.

**Methods:**

A total of 912 T2DM patients were enrolled. The urinary albumin-to-creatinine ratio (UACR) was measured in morning urine samples to assess DN. AGEs were non-invasively measured through skin autofluorescence. Recognizing the role of age in both AGEs and DN, AGE_age_ was calculated as AGEs × age/100 for related analyses.

**Results:**

The overall prevalence of DN in the present study was 37.2%. Elevated AGE_age_(χ^2^ = 61.06) was associated with a higher prevalence of DN. Multivariable linear regression demonstrated that AGE_age_ was positively associated with UACR levels(β = 0.154, 95% CI: 0.126, 0.306, P<0.001). In the moderation analysis, glycated hemoglobin A_1c_ (HbA_1c_) affected the correlation between AGE_age_ and UACR. Body mass index (BMI) and triglyceride glucose-body mass index (TyG-BMI) also affect the correlation between AGE_age_ and UACR, there were significant interactions between AGE_age_, HbA_1c_, BMI, TyG-BMI, and UACR.

**Conclusions:**

Complex associations and interactions were observed between AGEs, metabolic metrics, HbA_1c_, and DN. Implementing comprehensive interventions can potentially benefit the prevention of DN in T2DM patients.

## 1 Introduction

Type 2 diabetes mellitus (T2DM) has escalated into a global health crisis, which stands as the 11th leading cause of death worldwide due to chronic complications (1). Among the myriad microvascular complications associated with T2DM, diabetic nephropathy (DN) emerges as one of the most prevalent and severe, often culminating in end-stage kidney disease (ESKD). Current evidence suggests that DN is detected in approximately 33.6% of diabetic patients (2). It is generally characterized by an initial elevation in microalbuminuria excretion, a substantial increase in albuminuria, and a decline in glomerular filtration rate (GFR) (3). Research has underscored that diabetic patients exhibiting albuminuria are at a heightened risk of cardiovascular disease, mortality, and renal deterioration (4). Therefore, albuminuria serves as an early indicator of DN. Once DN manifests, its progression is challenging to reverse. Importantly, identifying diabetic patients prone to developing albuminuria could significantly aid in preventing the onset of DN.

Advanced glycation end products (AGEs) arise from the nonenzymatic glycosylation of proteins and lipids (5). This glycosylation process is intricate and slow. However, in a prolonged state of elevated glucose levels, glycosylation rates significantly accelerate, increasing AGEs. Studies have demonstrated a clear correlation between AGE accumulation in tissues and blood glucose levels (6). Furthermore, even after correcting hyperglycemia, AGE levels in diabetic tissues often fail to return to normal, leading to the concept of “metabolic memory” (7). Unlike HbA_1c_, AGEs are not merely byproducts of hyperglycemia but are also implicated in the development of diabetes (8). It is now understood that AGEs can crosslink with proteins, altering their structure, interfering with their functional properties, and binding to the receptor for advanced glycation end products (RAGE), thereby activating proinflammatory signaling pathways (9). These processes are also thought to contribute to the development of diabetic microvascular complications (10). Therefore, AGEs are gaining increasing attention, especially concerning their potential role as markers of DN. However, current methods for measuring AGEs are often complex and costly, making the need for cost-effective, portable, and stable measurement methods paramount. The non-invasive diabetes detector (DM scan), developed using optical detection technology for AGEs, offers the advantage of rapid, non-invasive measurements without the risk of cross-infection. Nevertheless, few studies have explored the relationship between DN and AGEs measured by skin autofluorescence.

While the significance of glycemic control in DN management has been established, it is imperative to consider other metabolism-associated risk factors. Obesity, a burgeoning global public health concern (11), has also been linked to kidney disease (12), with body mass index (BMI) serving as a common measure of obesity. A study in the UK revealed a positive correlation between higher BMI and an increased incidence of microalbuminuria, with this association particularly pronounced among individuals with higher BMI (13). Beyond BMI, various metabolic metrics are employed to assess their relationship with kidney disease. One such metric, the triglyceride-glucose-BMI (TyG-BMI) index, is a product of fasting blood glucose and triglyceride levels combined with BMI. It is currently used to evaluate the association with diabetes (14) and is considered an alternative surrogate marker for insulin resistance (IR), which itself is linked to kidney disease (15). However, few studies have investigated the association between TyG-BMI and DN.

As the prevalence of diabetes continues to surge, the burden of diabetes-associated nephropathy is also poised to increase. Accordingly, there is a pressing need for enhanced clinical prevention strategies to mitigate modifiable DN risk factors. Most existing studies have predominantly focused on the relationship between individual risk factors and DN, with few examining potential synergistic effects among these risk factors. Acknowledging the influence of glycemic management on DN, this study incorporates HbA_1c_ into the model. Accordingly, we put forth the following hypotheses: 1) AGEs are associated with DN, 2) Obesity can modulate this relationship, and 3) An interaction exists between AGEs, obesity, HbA_1c_, and DN. The outcomes of this study are anticipated to provide vital insights for healthcare providers and decision-makers, facilitating informed clinical decisions in the realm of healthcare.

## 2 Materials and methods

### 2.1 Study design and participants

Given the complexity of DN and the absence of a genetic or proteomic marker for accurate DN prediction, we opted to assess the modifiable risk factors for DN, thereby enabling more practical approaches to DN prevention and risk management. Most DN prediction models include non-modifiable factors such as age and disease duration (16, 17). While these factors influence DN, they are beyond our control. Therefore, we focused on intervenable and manageable risk factors in this study.

This cross-sectional study employed comprehensive survey procedures to investigate the impact of metabolic factors on albuminuria. We collected data from inpatients diagnosed with T2DM admitted to the Department of Endocrinology at First Affiliated Hospital of Anhui Medical University from September 1, 2019 to September 30, 2020. Through the patient’s hospitalization number, we were still able to identify individual participant information during or after data collection. The diagnosis of T2DM was based on the 1999 World Health Organization (WHO) criteria (18). The study received approval from the Ethics Committee of the First Affiliated Hospital of Anhui Medical University, and written informed consent was obtained from all participants (Ethics Committee approval number PJ2023-11-43).

### 2.2 Sample size estimation

Based on previous research indicating a 33.6% incidence of DN among diabetic patients (2) and the desired level of relative precision of 0.15(ϵ), α=0.05, Z_1-α/2_ = 1.96, the minimum sample size was determined to be 172 using the following formula. Considering the design of diabetic nephropathy staging, ensuring that each group had a certain sample size for stratified analysis, we investigated 940 patients.


$$n=\frac{(1-p)Z_{1-\alpha /2}}{\epsilon^{2}p}$$

### 2.3 Inclusion and exclusion criteria

We included patients with T2DM between 18 and 80 years of age. Exclusion criteria encompassed: (1) acute illnesses; (2) known genetic renal diseases; and (3) acute renal failure attributed to factors such as drug use or contrast agents. Of the 940 patients initially considered, 28 were excluded due to missing potential confounding factors, ultimately leaving us with a total of 912 T2DM patients included in the study.

### 2.4 Exposure

All participants underwent a comprehensive medical history assessment and physical examination, including age, diabetes duration, current hypoglycemic regimen, past medical history, height, weight, and blood pressure. Body Mass Index (BMI) was calculated as weight (kg)/height^2^(m^2^). Fasting venous blood samples were collected for laboratory assays, including fasting plasma glucose (FPG), HbA1c, total cholesterol (TC), triglycerides (TG), creatinine (Cr), and uric acid (UA).

Hypertension was defined as SBP ≥ 140 mmHg or DBP ≥ 90 mmHg or current use of antihypertensive medication (19). Hyperlipidemia was defined as TC>5.69 mmol/L or TG>1.68 mmol/L or current lipid-lowering medication use. According to Chinese criteria, overweight was defined as BMI ≥ 24 kg/m² and < 28 kg/m2, while obesity was defined as BMI ≥28 kg/m2 (20). HbA1c levels exceeding 7.0% were considered elevated (21). The age limit was set at 65 years based on the literature (22). The maximum diabetes duration was 10 years (22). The study utilized two surrogate markers of IR: TyG (23) and TyG-BMI (24). The estimation of the glomerular filtration rate (eGFR) was conducted through calculation using the Chronic Kidney Disease Epidemiology Collaboration (CKD-EPI) equation (25).

### 2.5 Outcome

Morning urine samples were collected to measure urinary albumin-to-creatinine ratio (UACR) levels. Albuminuria was categorized as nonalbuminuria (<30 mg/g), microalbuminuria (30 to 300 mg/g), or macroalbuminuria (>300 mg/g) (26).

### 2.6 Assessment of AGEs

Skin AGEs were assessed using the DM Scan detection device (Anhui Yikangda Optoelectronics Technology Co., Ltd., Hefei, China). The device employed an excitation light source with a peak wavelength of 370 nm to illuminate approximately 0.1 cm² of skin, measuring emitted light with a spectrometer within the range of 420 - 600 nm. Skin autofluorescence was calculated from the ratio of emitt**e**d light to reflected light using DM Scan software version 1.02. All measurements were conducted by trained nurses in semi-dark, room-temperature settings. Emphasis was placed on taking measurements from normal skin sites devoid of visible vessels, scars, lichenization, or other skin irregularities. Each subject’s skin AGEs were measured three times, and the mean was recorded. AGE_age_ was calculated as AGEs × age/100.

### 2.7 Sensitivity analysis

To assess the robustness of the model, we employed UACR as a categorical variable in the moderation analysis.

### 2.8 Statistical analysis

All data were subjected to statistical analysis using SPSS 23.0. Demographic and clinical characteristics of the participants were presented as either means with standard deviations or interquartile ranges (IQRs) for skewed data. Missing values were not filled in and were normally processed for analysis. The analysis proceeded through four distinct steps. Step 1 entailed the descriptive statistics, providing an overview of the general situation within the three albuminuria groups. Step 2 involved calculating Spearman’s correlation coefficients to assess the relationships between UACR and other biomarkers. Moving to Step 3, we conducted a multivariable logistic regression analysis to unveil the associations between metabolic indicators and UACR. Finally, in Step 4, we undertook a moderating analysis using the PROCESS method to elucidate the intricate relationships between metabolic indicators and UACR. To establish the presence of a moderating effect, the following criteria needed to be met: (a) a significant direct effect of AGE_age_ on UACR, (b) a significant direct effect of the moderator (metabolic metrics) on UACR, and (c) a significant direct interaction effect (AGE_age_ × HbA_1c_ × metabolic metrics) on UACR. Within SPSS software, the interactive effect was automatically computed, and it also provided the proportion of variance explained by the moderating effect of BMI (indicated by an increase in R^2^). A significant moderating effect was considered when the 95% confidence interval (CI) did not include zero.

## 3 Results

### 3.1 Characteristics of the study population

A total of 940 patients diagnosed with type 2 diabetes were initially enrolled in this study. After excluding those with missing data, the final analysis included 912 patients with T2DM (470 men and 442 women). The clinical characteristics of the participants, categorized based on the degree of albuminuria, are presented in **Table 1**. Notably, 339 participants exhibited higher levels of UACR, resulting in an overall prevalence of 37.2%. Among the various factors analyzed, older age (χ^2^ = 8.305), longer duration of diabetes (χ² = 35.284), higher systolic blood pressure (SBP) (χ^2^ = 60.268), diastolic blood pressure (DBP) (χ^2^ = 6.55),increased accumulation of AGEs (χ^2^ = 31.66), higher AGE_age_ (χ^2^ = 61.06), higher triglyceride (TG) levels (χ² = 8.716), higher total cholesterol (TC) levels (χ² = 14.362), Female gender (χ^2^ = 23.135), higher TyG (χ^2^ = 21.351), higher TyG-BMI (χ^2^ = 18.62), and lower eGFR (χ^2^ = 225.7) were significantly associated with a higher prevalence of albuminuria. Conversely, factors such as BMI(χ^2^ = 3.164), HbA_1c_ (χ^2^ = 2.484),did not exhibit significant correlations across the three groups.

**Table 1:** The prevalence characteristics of three groups of albuminuria.

		nonalbuminuria	microalbuminuria	macroalbuminurianormal	χ^2^value	P
Age					8.305*	0.016
	<65	437(65.2%)	173(25.8%)	60(9%)		
	≥65	136(56.2%)	71(29.3%)	35(14.5%)		
BMI					3.164	0.531
	Normal	257(64.6%)	103(25.9%)	38(9.5%)		
	Overweight	221(63.5%)	90(25.9%)	37(10.6%)		
	Obesity	94(57%)	51(30.9%)	20(12.1%)		
durations					35.284**	<0.001
	<10	344(69.8%)	122(24.7%)	27(5.5%)		
	≥10	228(54.5%)	122(29.2%)	68(16.3%)		
HbA_1c_					2.484	0.289
	<7	87(69%)	28(22.2%)	11(8.7%)		
	≥7	484(61.7%)	216(27.6%)	84(10.7%)		
DBP					6.55*	0.038
	Normal	461(64.7%)	177(24.8%)	75(10.5%)		
	Abnormal	111(56.1%)	67(33.8%)	20(10.1%)		
SBP					60.268**	<0.01
	Normal	404(72.1%)	122(21.8%)	34(6.1%)		
	Abnormal	168(47.9%)	122(34.8%)	61(17.4%)		
AGE					31.66**	<0.01
	≤P25	168(73%)	48(20.9%)	14(6.1%)		
	P25-P50	153(67.4%)	58(25.6%)	16(7%)		
	P50-P75	137(59.6%)	66(28.7%)	27(11.7%)		
	>P75	115(51.1%)	72(32%)	38(16.9%)		
AGE_age_					61.06**	<0.01
	≤P25	160	56	12		
	P25-P50	154	50	24		
	P50-P75	103	68	57		
	>P75	51	134	43		
TG					8.716*	0.013
	Normal	317(67.2%)	109(23.1%)	46(9.7%)		
	Abnormal	253(57.9%)	135(30.9%)	49(11.2%)		
TC					14.362**	0.001
	Normal	507(64.3%)	210(26.6%)	71(9%)		
	Abnormal	63(52.1%)	34(28.1%)	24(19.8%)		
Gender					23.135**	<0.001
	Male	323(68.7%)	118(25.1%)	29(6.2%)		
	Female	250(56.6%)	126(28.5%)	66(14.9%)		
TyG index					21.351**	0.002
	≤P25	161(70%)	52(22.6%)	17(7.4%)		
	P25-P50	151(66.8%)	51(22.6%)	24(10.6%)		
	P50-P75	144(62.9%)	60(26.2%)	25(10.9%)		
	>75	114(50.9%)	81(36.2%)	29(12.9%)		
TyG-BMI					18.62**	0.005
	≤P25	160(70.2%)	55(24.1%)	13(5.7%)		
	P25-P50	152(67%)	50(22%)	25(11%)		
	P50-P75	135(59.5%)	64(28.2%)	28(12.3%)		
	>75	123(54.2%)	75(33%)	29(12.8%)		
eGFR	≥90	427(70.9%)	156(25.9%)	19(3.2%)	225.7**	<0.001
ml/(min·1.73m^2^)	60-89	127(59.1%)	60(27.9%)	28(13.0%)		
	30-59	19(24.7%)	23(29.9%)	35(45.4%)		
	15-29	0	4(30.8%)	9(69.2%)		
	<15	0	1(20.0%)	4(80.0%)		

*P <0.05, **P <0.01.

### 3.2 Spearman correlation analysis between the risk factors and UACR

Next, spearman correlation analysis was utilized to assess the relationships between AGE_age_, BMI, TyG-BMI, HbA_1c_, and UACR. The results indicated that AGE_age_ exhibited a significant association with BMI (rs=-0.218, P<0.01), TyG-BMI (rs=-0.27, P<0.01), HbA_1c_ (rs=-0.103, P<0.01), and UACR (rs = 0.157, P<0.01). Additionally, BMI showed a significant correlation with TyG-BMI (rs=0.904, P<0.01) but did not exhibit statistically significant correlations with HbA_1c_ (rs = -0.03, P > 0.05) or UACR (rs=0.056, P>0.05). TyG-BMI demonstrated significant correlations with HbA_1c_ (rs=0.078, P<0.05) and UACR (rs=0.137, P < 0.01), while HbA_1c_ displayed a significant correlation with UACR (rs=0.094, P<0.01). The results are summarized in **Table 2**.

**Table 2:** The Spearman correlation matrices for AGE_age_, HbA_1c_, BMI, TyG-BMI, and UACR.

	AGE_age_	BMI	TyG-BMI	HbA_1c_	UACR
AGE_age_	–	-0.218**	-0.27**	-0.103**	0.157**
BMI	-0.218**	–	0.904**	-0.03	0.056
TyG-BMI	-0.27**	0.904**	–	0.078*	0.137**
HbA_1c_	-0.103**	-0.03	0.078*	–	0.094**
UACR	0.157**	0.056	0.137**	0.094**	–

*P <0.05, **P <0.01.

### 3.3 Multilevel linear regression between UACR and independent variables

In **Table 3**, the data indicated a dose-response relationship between AGE_age_ and UACR (β=0.154), There was a borderline dose-response relationship between HbA_1c_ and UACR (β=0.064). However, no dose-response relationship was observed between BMI, TyG-BMI, and UACR. After adjusting for gender and age, the relationship between AGE_age_, HbA_1c_ and UACR remained statistically significant. Notably, there was a dose-response relationship between BMI, TyG-BMI, and UACR (BMI: β=0.05, TyG-BMI: β=0.086).

**Table 3:** The multilevel linear regression between independent variables and UACR.

UACR							
AGE_age_	R^2^	β	t	P	F	LLCI	ULCI
Model 1	0.024	0.154	4.705	<0.001	22.138	0.126	0.306
Model 2	0.049	0.325	4.909	<0.001	15.756	0.274	0.639
HbA_1c_							
Model 1	0.004	0.064	1.918	0.055	3.678	-0.014	1.202
Model 2	0.03	0.076	2.305	<0.001	9.339	0.106	1.314
BMI							
Model 1	0.001	0.025	0.758	0.449	0.575	-0.235	0.531
Model 2	0.027	0.05	1.505	<0.001	8.278	-0.09	0.682
TyG-BMI							
Model 1	0.003	0.055	1.647	0.1	2.713	-0.005	0.059
Model 2	0.031	0.086	2.528	<0.05	9.793	0.009	0.075

Model 1: crude model; Model 2: Controlled for patients’ gender and age.AGE_age_, advanced glycation end products × age/100 index; HbA_1c_, glycated hemoglobin A_1c_; BMI, body mass index; TyG-BMI, triglyceride glucose-body mass index.

### 3.4 Moderation analysis

Moderation analyses were performed for AGE_age_, HbA_1c_, BMI, and UACR, as shown in **Table 4**. First, AGE_age_ significantly predicted the severity of UACR (P < 0.05). However, HbA_1c_ was not associated with UACR (P > 0.05), and BMI exhibited no significant correlation with UACR (P > 0.05). Second, the moderation analysis revealed that HbA_1c_ moderated the effect of AGE_age_ on UACR (P < 0.01). Similarly, BMI moderated the effect of AGE_age_ on UACR (P < 0.05), indicating that higher levels of both HbA_1c_ and BMI were associated with increased AGE_age_ and, subsequently, higher UACR levels. BMI did not moderate the effect of HbA_1c_ on UACR (P > 0.05). Finally, a significant three-way interaction among AGE_age_, BMI, and HbA_1c_ was observed for UACR levels in the overall sample (P < 0.01).

**Table 4:** Association between AGE_age_ and HbA_1c_, BMI and UACR.

Variables	UACR(continuity variable)					
	*coeff*	*SE*	*t* value	*P* value	LLCI	ULCI
AGE_age_	-3.0847	1.3348	-2.3109	0.0211	-5.7044	-0.4650
HbA_1c_	-7.1257	6.8073	-1.0468	0.2955	-20.4857	6.2344
BMI	-3.9661	2.7027	-1.4674	0.1426	-9.2705	1.3383
Int_1	0.1560	0.0559	2.7904	0.0054	0.0463	0.2658
Int_2	0.3277	0.1361	2.4076	0.0163	0.0606	0.5948
Int_3	0.4126	0.2790	1.4790	0.1395	-0.1349	0.9601
Int_4	-0.0156	0.0058	-2.7080	0.0069	-0.0269	-0.0043

Int 1: AGE_age_ × HbA_1c_; Int 2: AGE_age_ × BMI; Int 3: HbA_1c_ ×BMI; Int 4: AGE_age_ × HbA_1c_ × BMI; AGE_age_, advanced glycation end products × age/100 index; HbA_1c_, glycated hemoglobin A_1c_; BMI, body mass index.

Additional moderation analyses were conducted for AGE_age_, HbA_1c_, TyG-BMI, and UACR, as detailed in **Table 5**. The results revealed no significant correlation between UACR and AGE_age_ (P > 0.05), HbA_1c_ (P > 0.05), or TyG-BMI (P > 0.05). However, moderation analysis indicated that both HbA_1c_ and TyG-BMI moderated UACR as a result of AGE_age_ (P < 0.05), suggesting that elevated levels of HbA_1c_ and TyG-BMI were associated with increased AGE_age_ and subsequent elevations in UACR. Notably, TyG-BMI did not moderate the effect of HbA_1c_ on UACR (P > 0.05). Moreover, a significant three-way interaction among AGE_age_, TyG-BMI, and HbA_1c_ was observed for UACR levels in the overall sample (P < 0.05).

**Table 5:** Association between AGE_age_ and HbA_1c_, TyG-BMI and UACR.

Variables	UACR(continuity variable)					
	*coeff*	*SE*	*t* value	*P* value	LLCI	ULCI
AGE_age_	-1.5556	0.8933	-1.7414	0.0820	-3.3089	0.1976
HbA_1c_	-4.2932	4.8096	-0.8926	0.3723	-13.7325	5.1461
TyG-BMI	-0.2624	0.2058	-1.2754	0.2025	-0.6663	0.1414
Int_1	0.0102	0.0041	2.4531	0.0143	0.0020	0.0183
Int_2	0.1867	0.930	2.0069	0.0451	0.0041	0.3693
Int_3	0.0315	0.0212	1.4810	0.1390	-0.0102	0.0732
Int_4	-0.0011	0.0004	-2.4215	0.0157	-0.0019	-0.0002

Int 1: AGE_age_ × HbA_1c_; Int 2: AGE_age_ × TyG-BMI; Int 3: HbA_1c_ ×TyG-BMI; Int 4: AGE_age_ × HbA_1c_ × TyG-BMI. AGE_age_, advanced glycation end products x age/100 index; HbA_1c_, glycated hemoglobin A_1c_; TyG-BMI, triglyceride glucose-body mass index.

### 3.5 Sensitivity analyses

An analysis using UACR as a three-level categorical variable was performed to further examine the interactions. The results, presented in **Table 6** and **Table 7**, indicated a significant three-way interaction among AGE_age_, BMI, and HbA_1c_ for UACR in the overall sample (P = 0.0563), along with a significant three-way interaction among AGE_age_, TyG-BMI, and HbA_1c_ for UACR (P < 0.05).

**Table 6:** Association between AGE_age_ and HbA_1c_, BMI among three groups of albuminuria.

Variables	UACR (classified variable)					
	*coeff*	*SE*	*t* value	*P* value	LLCI	ULCI
AGE_age_	-0.0692	0.0434	-1.5959	0.1109	-0.1543	0.0159
HbA_1c_	-0.2547	0.2211	-1.1516	0.2498	-0.6886	0.1793
BMI	-0.1076	0.0878	-1.2252	0.2208	-0.2799	0.0647
Int_1	0.0035	0.0018	1.9226	0.0548	-0.0001	0.0071
Int_2	0.0079	0.0044	1.7780	0.0757	-0.0008	0.0165
Int_3	0.0130	0.0091	1.4313	0.1527	-0.0048	0.0308
Int_4	-0.0004	0.0002	-1.9109	0.0563	-0.0007	0.0000

Int 1: AGE_age_ × HbA_1c_; Int 2: AGE_age_ × BMI; Int 3: HbA_1c_ × BMI; Int 4: AGE_age_ × HbA_1c_ × BMI. AGE_age_, advanced glycation end products x age/100 index; HbA_1c_, glycated hemoglobin A_1c_; BMI, body mass index.

**Table 7:** Association between AGE_age_ and HbA_1c_, TyG-BMI among three groups of albuminuria.

Variables	UACR (classified variable)					
	*coeff*	*SE*	*t* value	*P* value	LLCI	ULCI
AGE_age_	-0.0514	0.0287	-1.7883	0.0741	-0.1078	0.0050
HbA_1c_	-0.2517	0.1547	-1.6275	0.1040	-0.5553	0.0518
TyG-BMI	-0.0106	0.0066	-1.6090	0.1080	-0.0236	0.0023
Int_1	0.0003	0.0001	2.2673	0.0236	0.0000	0.0006
Int_2	0.0062	0.0030	2.0666	0.0391	0.0003	0.0121
Int_3	0.0014	0.0007	1.9900	0.0469	0.0000	0.0027
Int_4	0.0000	0.0000	-2.2131	0.0271	-0.0001	0.0000

Int 1: AGE_age_ × HbA_1c_; Int 2: AGE_age_ × TyG-BMI; Int 3: HbA_1c_ ×TyG-BMI; Int 4: AGE_age_ × HbA_1c_ × TyG-BMI; AGE_age_, advanced glycation end products x age/100 index; HbA_1c_, glycated hemoglobin A_1c_; TyG-BMI, triglyceride glucose-body mass index.

## 4 Discussion

In this retrospective cross-sectional study, several key findings emerged. First, we observed a DN incidence of 37.2% among hospitalized T2DM patients, slightly higher than the rates reported in previous studies (2). Notably, among these DN patients, 58.4% had a BMI exceeding 24 kg/m², and only 11.5% had HbA_1c_ levels below 7%. This finding highlights the inadequacy of comprehensive T2DM management among this population. Second, our study revealed a significant correlation between AGEs and DN, with higher AGE levels indicating an increased risk of DN. Considering the influence of age on both AGEs and DN, we introduced the AGE_age_ index, which integrates AGEs and age. Lastly, we identified a three-way interaction among obesity, AGEs, and DN with HbA_1c_ in this regulatory relationship. These findings were supported by the results of sensitivity analyses, emphasizing their robustness.

Unlike diabetic macroangiopathy, diabetic microangiopathy is more closely associated with blood glucose, as evidenced in numerous large clinical studies (27, 28). Chronic hyperglycemia leads to increased oxidative stress, initiating the accumulation of AGEs in cells via activation through pathways such as the hexose pathway, polyol pathway, and protein kinase C, resulting in overexpression of RAGE and subsequent activation of various inflammatory cytokines (29). Studies on animals have indicated that inhibiting carboxymethyllysine (CML) may protect against DN progression (30), while young diabetic rats treated with AGEs precursors exhibit renal lesions similar to those seen in aged diabetic rats (31). AGEs are, therefore, crucial in DN development, and AGEs-generated markers can be harnessed to assess DN risk.

Recent studies have shown that non-invasive devices measuring skin AGE fluorescence can be used for diabetes screening, offering a simple and rapid approach (32). However, previous research on the association between non-invasive skin AGEs and diabetic complications has primarily focused on Caucasian populations, showing significant positive correlations between AGEs and diabetic vascular complications (33). Given the impact of skin tone on skin AGE levels, research on the relationship between AGEs and DN in Chinese diabetic populations remains limited. In this study, we employed UACR as a marker for DN to investigate the AGE-DN relationship. Given the significance of age in both AGEs and DN, we introduced the AGE_age_ index. We found that AGE_age_ levels were significantly elevated in DN, and after adjusting for factors including age, sex, and HbA_1c_, AGE_age_ remained positively correlated with UACR levels. This finding indicates that AGE_age_ influences UACR independently of HbA_1c_, underlining its value in assessing DN.

One of the management strategies for T2DM is lifestyle modification, including weight loss. A longitudinal study involving 369,362 participants aged 2-15 years indicated that a high percentage of T2DM patients were obese (47.1%), with only 4.33% having a normal BMI (34). This underscores the strong link between obesity and diabetes. Moreover, studies have independently identified BMI as a risk factor for DN (16). Large population-based investigations have corroborated the increased risk of nephropathy in individuals with both diabetes and obesity, and this risk remains elevated even after stringent glycemic control (35). This highlights the role of obesity in DN development, independently of blood glucose control. Overall, our study findings confirm the association of BMI with DN, emphasizing the importance of BMI control in T2DM management.

The interaction between BMI and AGEs has become a research hotspot. AGEs typically accumulate slowly through glycation processes, with hyperglycemia and hyperlipidemia accelerating AGE accumulation *in vivo* (36). Given that both hyperglycemia and hyperlipidemia are prevalent in obese individuals, it is reasonable to speculate that AGE levels are higher in obese patients, as supported by previous research (37). Our study consistently validated the association between BMI and AGE_age_. *In vitro* and animal experiments further supported this relationship, demonstrating that RAGE overexpression induces adipocyte hypertrophy (38) and that mice fed a high-fat high-AGE diet exhibit greater weight gain and more visceral fat compared with mice fed a high-fat low AGE diet for 6 weeks (39). Additionally, obese individuals often have less healthy dietary habits, consuming highly processed Western-style foods rich in exogenous AGEs, which can be absorbed into the bloodstream and accumulate in the body (40). Considering this interaction, we propose that AGEs interact with BMI to facilitate DN development. Our study validated this hypothesis, with moderating analysis showing that AGE_age_ interacts with BMI to increase the UACR. In contrast, HbA_1c_ and BMI did not exhibit a synergistic effect on DN risk, underscoring the greater importance of AGE_age_ in DN, with BMI exacerbating the condition. Although HbA_1c_ did not exert a moderating effect on BMI, we identified a three-way interaction between AGE_age_, HbA_1c_, BMI, and UACR, suggesting that patients with T2DM, especially those with higher AGEs, obesity, and HbA_1c_ levels, are at a heightened risk of urinary proteinuria. Effective management of HbA_1c_ and weight reduction can mitigate the impact of AGEs on UACR, emphasizing the importance of a comprehensive approach. On one hand, it involves strict blood glucose control to reduce HbA_1c_ levels and minimize endogenous AGE production. On the other hand, it necessitates dietary control to reduce the consumption of high-AGE foods, thereby decreasing the absorption of exogenous AGEs and lowering the risk of obesity.

While obesity is primarily linked to dietary factors, there are additional contributors to obesity, including IR. The development of IR is closely associated with obesity in a complex relationship, both being integral components of the metabolic syndrome. IR is a well-established risk factor for cardiovascular and cerebrovascular diseases and plays a significant role in DN. Animal studies have shown that mice fed a high-fat diet, resulting in obesity and IR, exhibit increased UACR levels and altered renal outcomes, indicating tubular dilation and interstitial vacuolation (41). Therefore, we examined another metabolic indicator, TyG-BMI, to represent IR. TyG-BMI, derived from the product of the TyG index and BMI, effectively reflects various metabolic processes in the body. Studies have previously established that elevated levels of TyG-BMI can heighten the risk of prediabetes, especially among non-obese individuals (42). Causality between TyG-BMI and the incidence of diabetes has been reported, particularly in non-obese populations (14). Nevertheless, the relationship between TyG-BMI and DN has received less attention. Our study provided hitherto undocumented evidence of a significant positive relationship between TyG-BMI and UACR, indicating that TyG-BMI is a potential risk factor for DN, possibly surpassing BMI’s significance. As a moderating variable, TyG-BMI exerts a distinct influence on the relationship between AGEs and UACR levels. Concurrently, *in vitro* and animal experiments suggest that AGEs can influence cellular insulin sensitivity and insulin secretion capacity (43, 44). This implies that non-obese type 2 diabetes patients, despite seemingly meeting BMI standards, should consider other metabolic factors since BMI fails to capture fat distribution, and abdominal obesity is more strongly associated with IR.

Herein, we established a retrospective model to assess the correlation between these metabolic indicators and DN. We unveiled the intricate interaction among AGEs, obesity-related metabolic metrics, and HbA_1c_, all associated with UACR levels. This underscores the significance of comprehensive diabetes management. Given that albuminuria in diabetic patients is largely preventable, effective management and treatment strategies should persist even after the onset of DN, aiming to retard disease progression. Comprehensive management awareness is imperative for diabetic patients, and early, timely interventions can substantially reduce the incidence of DN.

This study boasts several strengths, including its multilevel design and the inclusion of a substantial sample size. Furthermore, our study uniquely investigates DN by exploring the relationship between obesity and non-invasive AGEs, offering compelling insights into preventing proteinuria in type 2 diabetes mellitus. However, certain limitations should be acknowledged. First, in recent years, a subtype of DN has been proposed with low estimated glomerular filtration rate but without albuminuria, accounting for about 10.1% of diabetes patients (45), thus this subtype therefore needs to be studied to adjust the management strategy. Second, the cross-sectional nature of this study makes it challenging to establish causal relationships or confirm long-term clinical outcomes.

In conclusion, our study highlights the higher incidence of DN within the hospitalized T2DM population. We propose multifaceted management strategies to prevent DN in T2DM patients. Additionally, we introduce AGE_age_, a non-invasive measure of accumulated AGEs adjusted for age, as a promising approach for identifying patients at high risk of developing DN.

## Data Availability

The raw data supporting the conclusions of this article will be made available from the corresponding author on reasonable use.
